# Advances in CAR‐Engineered Immune Cell Generation: Engineering Approaches and Sourcing Strategies

**DOI:** 10.1002/advs.202303215

**Published:** 2023-10-31

**Authors:** Zhaozhao Chen, Yu Hu, Heng Mei

**Affiliations:** ^1^ Institute of Hematology Union Hospital Tongji Medical College Huazhong University of Science and Technology 1277 Jiefang Avenue Wuhan Hubei 430022 China; ^2^ Hubei Clinical Medical Center of Cell Therapy for Neoplastic Disease Wuhan 430022 China

**Keywords:** cancer immunotherapy, CAR engineering, cell sources, gene delivery, in vivo generations, off‐the‐shelf, synthetic biology

## Abstract

Chimeric antigen receptor T‐cell (CAR‐T) therapy has emerged as a highly efficacious treatment modality for refractory and relapsed hematopoietic malignancies in recent years. Furthermore, CAR technologies for cancer immunotherapy have expanded from CAR‐T to CAR‐natural killer cell (CAR‐NK), CAR‐cytokine‐induced killer cell (CAR‐CIK), and CAR‐macrophage (CAR‐MΦ) therapy. Nevertheless, the high cost and complex manufacturing processes of ex vivo generation of autologous CAR products have hampered broader application. There is an urgent need to develop an efficient and economical paradigm shift for exploring new sourcing strategies and engineering approaches toward generating CAR‐engineered immune cells to benefit cancer patients. Currently, researchers are actively investigating various strategies to optimize the preparation and sourcing of these potent immunotherapeutic agents. In this work, the latest research progress is summarized. Perspectives on the future of CAR‐engineered immune cell manufacturing are provided, and the engineering approaches, and diverse sources used for their development are focused upon.

## Graphical abstract

Recent progress in CAR‐engineered immune cells has significantly impacted personalized immunotherapy. Nevertheless, the cumbersome manufacturing process and limited cell sources of autologous CAR products have hurdled accessibility for patients. In this work, pioneering engineering approaches and sourcing strategies are delved into, illuminating their potential to amplify therapeutic efficacy and reshape the landscape of treatment for cancer and beyond.

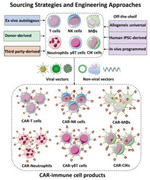

## 1 Introduction

Recent years have witnessed the emergence and rapid development of CAR technology, and CAR‐T immunotherapy has shown impressive clinical success for refractory and relapsed (r/r) hematopoietic malignancies, including CD19+ leukemia and lymphoma, as well as BCMA+ multiple myeloma.^[^ ^1^ ^]^ To date, nine CAR‐T products have been commercially approved worldwide for treating the above blood tumors.^[^ ^2^ ^]^ Motivated by what has been achieved, researchers have been expanding CAR technology from CAR‐T to CAR‐NK, CAR‐CIK, and CAR‐MΦ applications and employing CAR‐engineered cell therapy for wider indications to treat aggressive diseases.^[^ ^3^ ^]^ At present, 2313 clinical trials of CAR therapies have been carried out around the world.^[^ ^4^ ^]^ However, CAR therapy in solid tumors and hematological malignancies still faces serious challenges; there remains much room for optimization, including overcoming challenges such as limited immune cell sources and unstable quality of autologous immune cells;^[^ ^5^ ^]^ the labor‐intensive, time‐consuming and resource‐intensive manufacturing workflows;^[^ ^6^ ^]^ severe life‐threatening adverse reactions, including systemic hyperproduction of cytokines and neurologic toxicity; tumor recurrence induced by antigen escape; cell tolerance leading to restricted trafficking and limited tumor infiltration of CAR‐T cells; and the hostile and tumoricidal immunosuppressive tumor microenvironment (TME).^[^ ^7^ ^]^ Among these challenges, the limited immune cell supply and the complex ex vivo manufacturing process of autologous CAR products are issues that cannot be overlooked.

Obviously, autologous CAR products are derived from the patient's own immune cells, ensuring a personalized treatment approach tailored to individual needs, and there is no risk of graft‐versus‐host disease (GVHD). Nevertheless, the quantity and quality of immune cells are highly dependent on the immune cell status of cancer patients. It is reported that autologous T cells might be dysfunctional and exhausted in cancer due to suppressive TME.^[^ ^5^, ^8^ ^]^ In addition, for patients with T cell leukemia, the availability of high‐quality autologous T cells suitable for CAR‐T manufacturing is extremely limited.

Recently, various alternative engineering approaches and sourcing strategies have been developed for generating CAR‐engineered cells. In this review article, we will hold a detailed overview of the recent advancements in the current progress of gene‐delivering technology for manufacturing CAR‐modified immune cells and discuss these acquisition and engineering techniques, emphasizing the importance of proper sourcing and preparation of immune cells for the successful development of effective CAR‐based immune cell products.

## 2 Gene Delivery Technologies for CAR Engineering

**Scheme 1:**
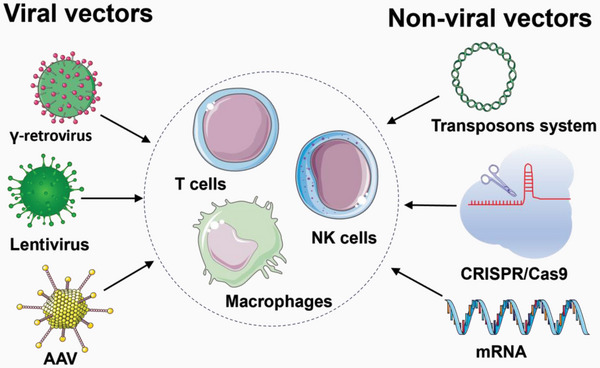
Gene delivery methods by viral vectors (γ‐retrovirus, lentivirus, and AAV) and non‐viral vectors (transposons system, CRISPR/Cas9, and mRNA) for immune cells CAR engineering.

For gene delivery approaches, viral transduction and non‐viral transfection have been used for CAR engineering (**Scheme** **1**).

### 2.1 Viral Vectors

Lentivirus, γ‐retrovirus and adeno‐associated virus (AAV) are the most commonly used viral vectors.^[^ ^9^ ^]^ It has been reported that ≈94% of evaluable CAR‐T products are prepared by viral vectors, with >50% mediated by lentiviral vectors.^[^ ^6^ ^]^ However, there exist several shortcomings hard to bypass for lentiviral vectors. First of all, in the process of CAR gene integration into T cells, lentiviral vectors often undergo random integration into the cellular genome, potentially causing adverse effects on the host genome, further leading to unintended gene silencing, overexpression, or genetic mutations, thereby increasing potential safety risks.^[^ ^10^ ^]^ What is more, the limited transcriptional capacity of lentiviral vectors restricts the size and complexity of the CAR gene payloads and their associated regulatory elements that can be accommodated.^[^ ^11^ ^]^ Last but not least, the large‐scale production of lentiviral vectors for clinical applications requests matching good manufacturing practice (GMP)‐graded laboratory and manufacturing reagents, resulting in close‐to‐prohibitive manufacturing costs and regulatory hurdles.^[^ ^12^ ^]^ Therefore, it is imperative to develop safer and lower‐cost viral‐free gene delivery vectors, typical of which are transposon systems, clustered regularly interspaced short palindromic repeats (CRISPR)/Cas9 systems and mRNA electroporation platforms (**Table** **1**).^[^ ^13^ ^]^

**Table 1:** Comparison of viral and non‐viral vectors for CAR engineering.

Viral vector		Nonviral vector		
γ‐Retrovirus	Lentivirus	Transposon	CRISPR/Cas9	mRNA
Production	Labor‐intensive	Simple	Moderate	Easy
Transfection efficiency	Low to medium	Various	High	Various/high
Genome integrity	Yes	Yes	Yes	None
Insertion sites	Random	Not completely random	Site‐specific	None
Payload capacity	≈8 kb	≈10 kb (SB)/≈220 kb (PB)	Moderate	Large
Stability	Stable	Stable	Stable	Transient
Mutagenesis risk	High	Medium to low	Very Low	None
Overall cost	Very costly	Cheap	Cheap	Cheap

### 2.2 Non‐Viral Vectors

#### 2.2.1 Transposon System

**Scheme 2:**
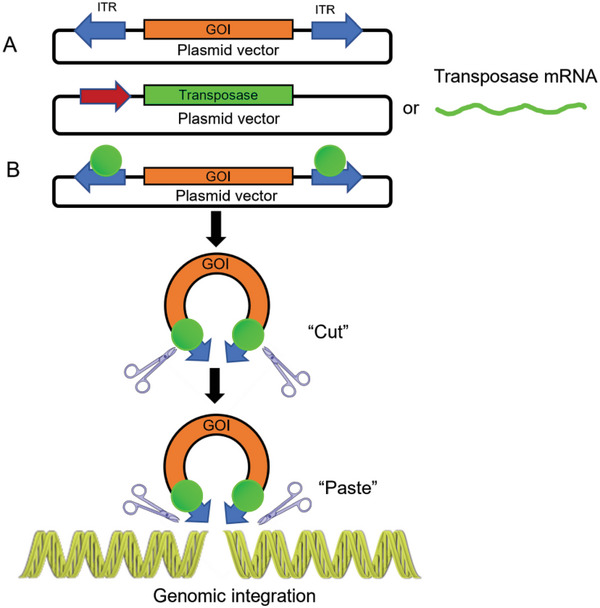
Schematic diagram of genomic integration mode for SB transposon system. A) Construction of transposon plasmid vector encoding the GOI and inverted terminal repeat (ITR), a specific sequence that could bind transposase at both ends of GOI, and construction of pDNA or mRNA encoding transposase. B) Gene delivery mechanism: Once expressed inside the host cell, the transposase will bind to the ITR sequences at both sides of GOI and excise the GOI from the transposon. Subsequently, the excised GOI‐transposase complex will actively search for genomic regions rich in TA sequences, facilitating the integration of the GOI into the host genome.

The transposon system consists of two main components: the transposase and the transposon. The transposase recognizes and cleaves specific sequences in the transposon DNA, catalyzing its excision or integration, and the transposon contains the movable gene segment, which carries the gene of interest (GOI) and specific sequences required by the transposase. By undergoing excision and integration within the host genome, the transposon system can achieve rearrangements at different sites through “cut‐and‐paste” or “copy‐and‐paste” mechanisms^[^ ^13^, ^14^ ^]^ (**Scheme** **2**). Common transposon systems involve Sleeping Beauty (SB), piggyBac (PB), and Tol2.^[^ ^15^ ^]^ For CAR engineering, a plasmid DNA (pDNA)/mRNA or dual‐plasmid system of the art in vectorology is usually employed, wherein the GOI and the transposase are encoded separately, allowing for adjusting their ratio of them to optimize the transfection efficiency. Reportedly, SB transposon system via a combination of minicircle transposon DNA and transposase mRNA in therapeutic gene transfer to human hematopoietic stem cells has previously been validated.^[^ ^16^ ^]^ As a representative, this art in vectorology has also been highly regarded for CAR gene integration in human T cells, enabling enduring gene expression, thus facilitating the swift generation of CD19‐CAR T‐cells, and empowering robust antitumor capabilities in a xenograft model.^[^ ^17^ ^]^ Also, Harjeet Singh and his coworkers presented an approach involving the direct introduction of dual‐plasmid DNA derived from the SB system to express a CD19‐CAR in both memory and effector T cells by electroporation, entirely circumventing the need for drug selection.^[^ ^18^ ^]^ In addition, PB transposon system as a novel methodology for the efficient generation of CAR‐Ts has also earned widespread interest in recent years,^[^ ^19^ ^]^ due to the fact that the superiority of PB over SB lies in its capability to integrate a larger gene payload capacity (∼200 kb compared to ∼10 kb)^[^ ^20^ ^]^ and achieve more precise excision, leading to higher transposition activity.^[^ ^13a^ ^]^ Other CAR‐modified immune cells on the basis of the transposon system including CAR‐NK^[^ ^21^ ^]^ and CAR‐CIK^[^ ^22^ ^]^ have been enclosed in exploration as well.

Remarkably, virus‐free transposon systems offer several advantages over viral‐based gene delivery methods: 1) manufacturing complexity and production costs: The transposon systems typically involve the direct delivery of pDNA or mRNA into host cells, avoiding the complexities associated with viral vector production, which relies on expansion of viral packaging cell lines, purification, sterile filtration, final product formulation, and storage, taking 2–3 weeks generally.^[^ ^23^ ^]^ In contrast, the production of plasmids uses an easy‐to‐culture and cost‐effective E. coli system, and the subsequent purification process is also simple and takes as little as 7–10 days under GMP conditions.^[^ ^23^ ^]^ In addition, the manufacturing of in vitro transcribed mRNA (IVT mRNA) is also straightforward and cost‐efficient;^[^ ^24^ ^]^ 2) stability and distribution of integration sites: compared to insertional viral vectors whose integration sites randomly distribute in a genome‐wide scale, transposons display a not completely random but relative specific integration pattern, in which SB and PB transposons were integrated into TA and TTAA‐enriched regions respectively in the host genome to induce stable expression of the GOI, significantly reducing the impact on the expression of a gene near insertion sites;^[^ ^15^ ^]^ 3) overall safety: the transposons are generally considered safer than viral vectors, as they do not carry the risk of generating replication‐competent viruses or eliciting immune responses against viral components. They are non‐infectious and would not impose threats to the environment, staff, or patients, and the potential for accidental release or exposure to infectious agents is minimized.

#### 2.2.2 CRISPR/Cas9 System

**Scheme 3:**
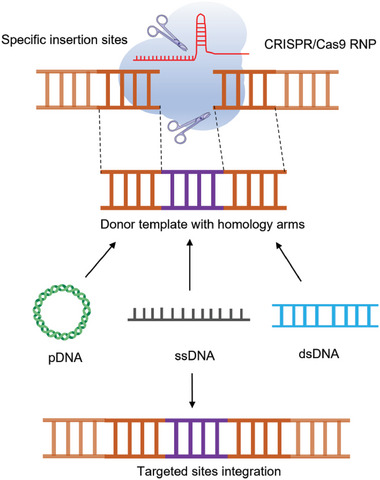
Overview of CRISPR/Cas9‐mediated knock‐in of the GOI integration. pDNA, single‐stranded DNA (ssDNA), and double‐stranded DNA (dsDNA) could serve as donor templates with homology arms. Under the guidance of the guide RNA (gRNA), the ribonucleoprotein (RNP) complex effectively targets a specific DNA sequence. Following nuclease binding, the DNA undergoes cleavage, initiating the subsequent repair process mediated by either non‐homologous end joining (NHEJ) or precise homology‐directed repair (HDR).

Other approaches inducing enduring and transient expression of CAR have also been inspected recently. CRISPR/Cas9 system achieves precise gene editing by introducing Cas9‐gRNA complexes into the genome, which induce double‐strand break (DSB) in targeted cells and exploit the host cell's endogenous DNA repair mechanism for accurate gene editing^[^ ^25^ ^]^ (**Scheme** **3**). Huang et al. demonstrated feasibility in a preclinical study by specifically inserting an anti‐CD19 CAR sequence into the programmed cell death protein 1 (PD1) gene based on the CRISPR/Cas9 systems by electrotransfection, thus inducing PD1 interference, and further enhancing the anti‐tumor activity.^[^ ^26^ ^]^ This group further validated their excellent safety and efficacy in treating patients with r/r B cell non‐Hodgkin lymphoma (B‐NHL).^[^ ^26^ ^]^

Concerningly, the application of CRISPR/Cas9 technology has raised potential safety issues. The foremost consideration centers on the potential risk of off‐target mutations, given the vast genome harboring multiple DNA sequences that bear identical and highly homologous to the target DNA sequence. Apart from the intended target DNA, the CRISPR/Cas9 system can inadvertently cleave these off‐target DNA sequences, resulting in unintended mutations at non‐targeted sites.^[^ ^25^, ^27^ ^]^ Such off‐target effects necessitate meticulous evaluation and validation to ensure the precision and safety of CRISPR/Cas9‐mediated gene editing, particularly when exploring therapeutic applications in medical research, exemplified by CAR immunotherapy. Posteriorly, because of that the CRISPR/Cas9 system is derived from bacteria and archaea,^[^ ^28^ ^]^ its application in gene editing may generate foreign immunogenic substances, thereby provoking a certain degree of immunogenicity risk.

#### 2.2.3 mRNA‐Based Platform

The utilization of mRNA technology for CAR product generation is a promising approach in immunotherapy, on account of circumventing the risk of insertional mutagenesis and allowing for a more rapid and flexible manufacturing process.^[^ ^24^ ^]^ One group evaluated preclinically, Descartes‐08, a CD8+ CAR‐T product on the basis of mRNA, to treat multiple myeloma, which expressed anti‐BCMA CAR for 7 days, demonstrating limited side effects of uncontrolled proliferation and diminishing production of inflammatory cytokines in response to target cells.^[^ ^29^ ^]^

### 2.3 The Safety Aspects of the CAR Construct

Ultimately, the safety implications inherent to the CAR construct necessitate comprehensive consideration and in‐depth discourse. In regard to CAR construct, multiple iterations have transpired over recent decades, with the second and third‐generation CAR constructs being the most prevalent in preclinical and clinical investigations.^[^ ^30^ ^]^ These constructs encompass an antigen‐binding domain (single‐chain Variable Fragment, scFV), a hinge region, a transmembrane domain, one co‐stimulatory domain, and an intracellular signaling domain, among which, scFv and co‐stimulatory domains have played pivotal roles in the process of antigen recognition and activation, respectively.^[^ ^31^ ^]^

To begin with, the consideration of immunogenicity stands as a paramount imperative. Mouse‐derived monoclonal antibodies (mAbs) generated through hybridoma technology exhibit pronounced immunogenicity,^[^ ^32^ ^]^ potentially rendering murine scFVs as antigenic entities within the human immune system due to differences in species, thereby instigating rejection responses and even precipitating severe immune‐related disorders. Consequently, the full humanization of scFvs emerges as a pivotal strategy,^[^ ^33^ ^]^ contributing to the enhancement of the safety and therapeutic potential of CAR‐immune cell therapies.

Moreover, the affinity of scFV and the choose of co‐stimulation molecules have a great impact on the tumoricidal efficacy, activation levels, and in vivo fate of CAR‐immune cells, resulting in varying degrees of CAR therapies‐associated toxicity. It was reported that high‐affinity CAR would induce significant activation‐induced cell death (AICD), impairing CAR‐T cell proliferation. Conversely, CARs with intermediate to low affinity may mitigate antigen loss triggered by trogocytosis, thereby engendering superior antitumor functionality.^[^ ^34^ ^]^ The CD28 co‐stimulatory domain mediates potent cytotoxicity, facilitates IL‐2 secretion, and favors the expansion of CD4+ T cells, in contrast to which, 4‐1BB tends to stimulate the generation of CD8+ central memory T cells, enhancing the persistence of CAR‐T cells consequently.^[^ ^35^ ^]^ Besides, clinical data from numerous CAR‐T therapies targeting B‐cell malignancies have confirmed that in comparison to 4‐1BB CAR‐T, CD28 CAR‐T cells are more prone to eliciting severe cytokine release sundrome (CRS) and immune effector cell‐associated neurotoxicity syndrome (ICANS).^[^ ^36^ ^]^ So, through the optimization of CAR structure, a harmonious interplay between scFV affinity and appropriate co‐stimulatory molecules can be orchestrated, enhancing specificity, affinity, and cytotoxicity equilibrium, as a consequence of which, this amplifies the anti‐tumor effect and enhances the safety profile of the therapy.

Eventually, the truncated human epidermal growth factor receptor (tEGFR) is generally inserted into the CAR structs that can be employed for ex vivo screening, flow cytometry, in vivo tracking, and a “safety switch” to eliminate CAR‐T cells in the body with Cetuximab,^[^ ^37^ ^]^ improving the convenience of CAR‐Ts detection and the safety of this treatment. Nevertheless, while tEGFR is incapable of binding to EGF and initiating EGFR signaling, being an exogenous molecule not naturally present in the human body, the extracellular tEGFR might activate the immune system, eliciting antibody responses and potentially leading to host rejection of CAR‐Ts.

## 3 Ex Vivo Autologous CAR‐Engineered Immune Cells

**Scheme 4:**
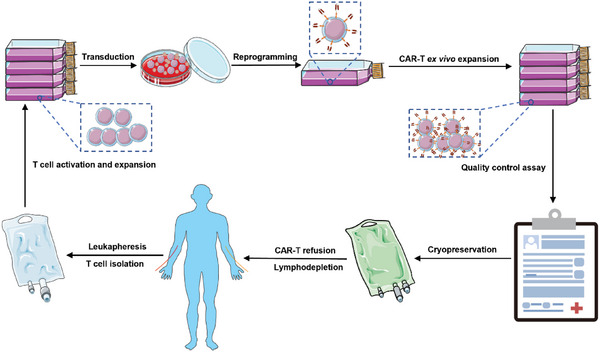
Schematic diagram of traditional ex vivo manufacturing process of autologous CAR‐T cells. This workflow entails leukapheresis and subsequent T cell isolation, activation, and expansion, followed by lentivirus transduction, then CAR‐T cells are amplified to make a cell formulation cryopreservation, and finally transfused to cancer patients supposed to receive lymphodepletion chemotherapy. In each batch especially before cryopreservation and refusion, quality control assay with utmost stringency should be implemented.

Traditionally, the autologous CAR‐T manufacturing and therapy workflow is relatively standardized and includes the following steps: 1) leukapheresis to isolate and enrich T cells from cancer patients; 2) T‐cell activation and expansion; 3) transduction of T cells with a CAR gene vector utilizing viral or nonviral systems; 4) ex vivo CAR‐T‐cell expansion; 5) packaging of cell product formulations and cryopreservation; and 6) administration of lymphodepleting treatment and subsequently reintroduction of CAR‐T cells in patients (**Scheme** **4**). Other CAR‐engineered immune cells (CAR‐NK, CAR‐MΦ, and CAR‐CIK) are prepared in a similar logistics.^[^ ^38^ ^]^ During these processes, as a “living” cellular drug, stringent quality control assays and release criteria to guarantee the integrity of the final CAR‐T products are needed, in which control of production materials (especially T cell sources and gene modification vectors), in‐process control and testing, release testing, validation of the production process, and stability study involving level of CAR expression, lymphocyte subpopulations, cell purity, number and ratio of living cells, in vitro potency, microbiological safety (sterility test, mycoplasma examination, replication‐competent virus detection, rapid microbiological detection, endotoxins, and etc.).^[^ ^39^ ^]^

The drawbacks of the in vitro generation of CAR products are obvious: 1) the whole process generally takes 7–22 days, and a GMP‐graded laboratory must be used; 2) the lengthy ex vivo culture conditions can remarkably impact CAR‐T‐cell functionality,^[^ ^40^ ^]^ and there are additional difficulties during the preparation of each batch, such as contamination of isolated T cells with red blood cells, granulocytes and/or platelets, as well as tumor cells; 3) variable T‐cell transfection efficiency; 4) contamination risk of in vitro cell expansion and effects of cryopreservation and transport on cell activity^[^ ^39a^ ^]^; 5) the highly technically demanding and labor‐intensive production process makes the therapy very expensive. As a consequence, many patients with corresponding indications cannot afford treatment, thus missing the optimal treatment time; 6) for patients who have undergone allogeneic hematopoietic stem cell transplantation, the composition of the T‐cell receptor Vβ repertoire in the early post‐transplantation phase might be subjected to remodeling due to immune reconstruction.^[^ ^41^ ^]^ Specific subtypes of T‐cell receptor Vβ repertoire would be impacted, potentially leading to reduced diversity of CAR‐T cells. This alteration in the T‐cell repertoire may have implications on the therapeutic efficacy of CAR‐T cells and their anti‐tumor capabilities; 7) in some cancer patients, particularly those with advanced disease, a considerable fraction of non‐chemo‐naïve T cells may have encountered tumor antigens or experienced repetitive activation and expansion, resulting in a state of exhaustion.^[^ ^42^ ^]^ These exhausted T cells may exhibit diminished responsiveness to reprogramming with CARs and impaired anti‐tumor functions, potentially compromising the therapeutic efficacy of the final CAR‐T products.

## 4 Donor‐derived CAR‐Modified Products

Given that autologous CAR products rely highly on sufficient endogenous immune cells, cancer patients who have suffered from lymphopenia or relapse after allogeneic hematopoietic stem cell transplantation (alloHSCT) may set out to exploit other cell sources. Recent advances provide an impressive breakthrough in the use of donor‐derived CAR products, and administration of CAR‐engineered immune cells derived from human leukocyte antigen (HLA)‐identical or HLA‐haplotype matched donors may save these issues (**Table** **2**).

**Table 2:** Representative donor‐derived CAR‐T products in clinical evaluation.

Disease	Cases	Donor type	CRS	GVHD	Outcome	Reference
CD19+ B‐ALL	6	Haplo‐	83.33%	33.33%	83.33% CR	[46]
CD19+ B‐ALL	43	Identical‐ (17), haplo‐ (26)	88%	4.65%	79% CR	[47]
CD19+ B‐ALL	6	Haplo‐	83.33%	None	83.33% CR	[49]
CD19+ B‐cell malignancies	20	Identical‐ (12), haplo‐ (8)	50% (≥3)	10%	40% CR or PR	[50]
CD7+ T‐ALL, T‐LBL	12	Identical‐ (1), haplo‐ (11), matched unrelated (1)	100%	50%	91.7% CR	[48]
CD7+ T‐ALL	20	Identical‐ and haplo‐	90%	60%	90% CR	[51]

First of all, CAR‐T cells derived from healthy donor volunteers exhibit enhanced T‐cell fitness and greater cancer‐killing capacity compared to autologous CAR‐T counterparts in cancer patients.^[^ ^43^ ^]^ Moreover, healthy donor CAR‐T cells demonstrate robust graft‐versus‐leukemia (GVL) efficacy with reduced detrimental GVHD activity,^[^ ^44^ ^]^ independent of the cytotoxic effects of CAR‐Ts.^[^ ^45^ ^]^ This potent GVL effect highlights the therapeutic potential of donor‐derived CAR‐Ts, offering a promising avenue for safer and more effective immunotherapies in the treatment of leukemia and other malignancies. Chen et al. reported the preliminary clinical outcomes of donor‐derived CD19 CAR‐T therapy in 6 patients with relapsed B‐cell malignancies who showed no response to donor lymphoblastic infusion (DLI) following HSCT, in which 5 patients (83.33%) achieved minimal residual disease (MRD)‐negative remission.^[^ ^46^ ^]^ Zhang et al. conducted an evaluation of donor CD19 CAR‐T therapy in 43 patients with relapsed CD19+ B‐cell acute lymphoblastic leukemia (B‐ALL) following allo‐HSCT.^[^ ^47^ ^]^ This study demonstrated the safety and efficacy of this approach, with 34 patients achieving complete remission (CR). Additionally, nine subjects experienced ≤ Grade 2 ICANS, while two participants exhibited ≤ Grade 2 acute GVHD. Li and her group employed a novel therapeutic approach involving donor‐derived CD7 CAR‐T therapy followed by allo‐HSCT from the same donor to treat 12 patients with r/r T‐cell acute lymphoblastic leukemia (T‐ALL) or T‐cell lymphoblastic lymphoma (T‐LBL), and 11 patients were alive and disease‐free at their last follow‐up.^[^ ^48^ ^]^

Furthermore, precious study revealed an anti‐leukemic effect of allogenic NK cells in allo‐HSCT,^[^ ^52^ ^]^ and donor‐derived memory‐like NK cells showed persistent function and induced remissions in leukemia patients following HSCT reportedly.^[^ ^53^ ^]^ Interestingly, due to the limited in vivo proliferation capacity, CAR‐NK therapy generally poses minimal risk of CRS and ICANS, and allogeneic CAR‐NK cell‐based adoptive cell therapy is not associated with GVHD.^[^ ^3^, ^54^ ^]^ Inspired by these advantages, healthy donor‐derived CAR‐NK cells would play an “off‐the‐shelf” role in cell sources. Several clinical trials harnessing donor CD19+ CAR‐NK immunotherapy for treating B‐cell malignancies have been carried out.^[^ ^55^ ^]^

## 5 Third Party‐Derived CAR‐Engineered Immune Cells

Third‐party‐derived CAR‐immune cells refer to cells genetically modified from a non‐HLA‐matched donor, umbilical cord blood (UCB)‐derived cells, immortalized cell lines in this review article, on behalf of alternative cell sources.

### 5.1 Non‐HLA‐Match Donor CAR‐Immune Cells

In contrast to donor‐derived CAR‐T cells, in the context of non‐HLA‐matched donor CAR‐immune cells, the foremost concern is to diligently avoid the occurrence of GVHD. αβ T cells, constitute ≈90% of the circulating T cells^[^ ^56^ ^]^ and are the main subtype of autologous CAR‐T cells. However, this particular T cell subset plays a leading role in the pathogenesis of acute and chronic GVHD.^[^ ^57^ ^]^ Hence, is imperative to employ gene editing strategies to disrupt the T cell receptor (TCR) and reduce the risk of GVHD occurrence, this point will be discussed in “Off‐the‐shelf CAR‐based agents” in this review. Another T cell subtype, γδT cells, have been highly favored recently, owing to the fact that they do not contribute to GVHD.^[^ ^58^ ^]^ Donor‐derived γδT cells utilize their intrinsic receptors to recognize a variety of tumor antigens in a histocompatibility complex (MHC) in an independent manner, allowing their application in an allogeneic setting without the need for TCR depletion. Furthermore, upon activation, they can elicit adaptive immune responses.^[^ ^58^, ^59^ ^]^ Correspondingly, clinical evaluation of CAR‐γδT cells in the treatment of solid tumors and hematological malignancies has bloomed everywhere.^[^ ^58^ ^]^

### 5.2 UCB‐Derived CAR‐Immune Cells

UCB stands as a pivotal and unparalleled reservoir of stem cells residing within the umbilical cord and placental matrix subsequent to parturition.^[^ ^60^ ^]^ These stem cells are conspicuously characterized by their extraordinary pluripotent capacity to undergo lineage‐specific differentiation encompassing hematopoietic, neural, and immunogenic lineages.^[^ ^61^ ^]^ Also, UCB represents an affluent repository harboring not only hematopoietic stem cells but also a profusion of immune effectors, prominently T cells and NK cells.^[^ ^62^ ^]^ This discerning reservoir thus has emerged as a propitious wellspring, offering prospective foundational materials for the development of CAR‐T and CAR‐NK cell‐based therapeutic strategies.^[^ ^63^ ^]^ In contradistinction to adult peripheral blood (PB), immunocytes within UCB show a greater degree of immaturity, reduced differentiation status, augmented proliferative potential, and a higher proportional representation of NK cells (15–30%).^[^ ^64^ ^]^ Blandine et al suggested that UCB‐derived CAR‐T cells exhibited a higher proportion of preserved T stem cell memory (T_SCM_) and central memory T cells (T_CM_) in comparison to their PB counterparts,^[^ ^62^, ^65^ ^]^ augmenting the potential for enhanced in vivo persistence and prolonged immune responses, thereby fostering a more enduring immunotherapeutic impact. An additional salient consideration lies in that UCB‐derived transplants demonstrate a diminished proclivity toward GVHD,^[^ ^66^ ^]^ signifying a mitigated risk in this regard.

Investigators have commenced the assessment of the viability of CAR‐immune cells originating from this specific cellular reservoir. In their research, Liu and colleagues harnessed the potential of a retroviral vector carrying CD19‐CAR, IL‐15, and an inducible caspase‐9 suicide gene (iC9) for the transduction of NK cells derived from UCB.^[^ ^67^ ^]^ The results of their investigation compellingly establish the efficacy of these modified NK cells in the specific targeting and eradication of CD19‐expressing cellular populations, including primary leukemia cells. In the xenotransplantation model utilizing Raji lymphoma‐inflicted mice, these UCB‐CAR‐NK cells exhibited a remarkable and substantiated elongation in overall survival rates. Yu et. al fabricated CD19 CAR‐Ts from UCB and subsequently evaluated the oncolytic potential preclinically in the context of diffuse large B cell lymphoma (DLBCL).^[^ ^68^ ^]^

### 5.3 Immortalized Cell Lines‐Derived CAR Products

Immortalized cell lines refer to cells that have been modified to evade the natural processes of senescence and death. These cell lines can replicate indefinitely under laboratory conditions, providing a consistent and potentially unlimited source of cells for various applications. NK‐92 is an IL‐2‐dependent NK cell line derived from peripheral blood mononuclear cells (PBMC) of a 50‐year‐old Caucasian male with aggressive NHL, exhibiting remarkable cytotoxic activity against a diverse range of malignant cells.^[^ ^69^ ^]^ Genetic modification through CAR engineering has the potential to enhance the tumor antigen‐specific recognition of NK‐92 cells, thereby enhancing their activation and consequently elevating the immune response. Michael et al. devised CAR‐NK‐92 cells that secrete IL‐15 against CD123+ acute myeloid leukemia (AML).^[^ ^70^ ^]^ The CAR‐NK‐92 cells demonstrated potent cytotoxicity in in vitro assessments, and within the peripheral blood of patient‐derived xenograft (PDX) models, they effectively eliminated CD123+ AML cells. Another research group presented preclinical evidence of CAR‐NK‐92 therapy for solid tumors. In this study, they developed mesothelin (MSLN)‐specific CAR‐NK‐92 cells for the treatment of gastric cancer, markedly extending the survival duration in murine models bearing intraperitoneal tumors.^[^ ^71^ ^]^ In clinical settings, Tang et. al conducted a first‐in‐man clinical trial to evaluate the safety profile of CD33‐CAR‐NK‐92 cells in individuals afflicted with r/r AML. Administered at dosages reaching 5 × 10^9 cells per patient, no notable adverse events, including severe CRS, were discerned.^[^ ^72^ ^]^

Nevertheless, it is regrettable that, due to their initial derivation from lymphoma patients,^[^ ^73^ ^]^ CAR‐NK‐92 cells require irradiation prior to infusion to curtail excessive in vivo expansion. While retaining a fraction of their specific cytotoxic potential, the in vivo proliferative capacity experiences a precipitous decline, leading to cellular depletion within 7 days post‐infusion.^[^ ^64^, ^72^ ^]^ Hence, repeated administration is needed to sustain therapeutic efficacy. In the future, alternative strategies such as administrating small molecule compounds, antibodies, or pre‐transducing suicide genes may potentially eliminate CAR‐NK‐92 cells within the body without compromising therapeutic efficacy, thereby addressing this limitation.

In the aspect of other CAR‐immune cells, two preclinical studies generated CAR‐MΦ from murine macrophage cell lines. Raw264.7 cells are derived from the ascites of an Abelson murine leukemia virus‐induced tumor in a male BALB/c mouse, displaying a spectrum of biological activities that render it a prominent tool for simulating and investigating diverse functionalities and responses inherent to macrophages.^[^ ^74^ ^]^ Researchers devised HER2‐CAR‐modified Raw264.7 cells, which, upon co‐culture with HER2+ human breast cancer cells, effectively triggered the expression of matrix metalloproteinases (MMPs) within macrophages. While not inhibiting tumor cell growth in vitro, intravenous administration of these CAR‐MΦ significantly suppressed tumor growth in a 4T1 breast cancer murine model. Additionally, an elevated proportion of T cells within tumors post‐infusion suggested that HER2‐CAR‐Raw264.7 disrupted the tumor extracellular matrix, facilitating deep infiltration of T cells into the TME.^[^ ^75^ ^]^ Alternatively, J774A.1 cell line also serves as a valuable macrophage model.^[^ ^76^ ^]^ In 1968, researchers from the National Cancer Institute first isolated the J774A.1 cell line from a macrophage tumor of a female BALB/cN mouse. Following cultivation and subsequent passages, this cell line was established as a stable and perpetuating cell lineage. Meghan and colleagues orchestrated the genetic modification of J774A.1 cell by integrating a spectrum of CARs for Phagocytosis (CAR‐Ps), guiding macrophages toward the engulfment of designated targets, encompassing cancer cells among others.^[^ ^77^ ^]^ Unfortunately, owing to interspecies disparities, these cell lines are not amenable to clinical CAR‐MΦ immunotherapy. Therefore, the prospective advancement of immortalized macrophage cell lines suitable for human applications holds promise in broadening the cellular reservoir for CAR‐MΦ sourcing. For example, further evolution and engineering of THP‐1 cells (a human monocytic cell line simulating monocytes/macrophages) may present itself as a favorable candidate.

## 6 Off‐the‐shelf CAR‐Based Agents

Recent strides in the realm of immunotherapy have yielded a pivotal breakthrough through the advent of off‐the‐shelf CAR‐engineered immune cells.^[^ ^78^ ^]^ This innovation heralds a paradigm shift, particularly in the therapeutic landscape of diverse ailments, prominently underscored by oncological afflictions.^[^ ^79^ ^]^ In stark contrast to conventional immunotherapeutic modalities necessitating autologous cell engineering bespoke for individual patients, these off‐the‐shelf CAR‐engineered immune cells have undergone pre‐engineering, rendering them promptly deployable for immediate intervention. Notable advantages accompany this groundbreaking progression. Foremost, it expedites the laborious and financially onerous custom‐tailoring process entailed in patient‐specific immune cell engineering, thereby expediting the commencement of therapeutic regimens. Furthermore, it surmounts impediments associated with the procurement and quality control of patient‐sourced cells. The establishment of a uniform manufacturing framework for off‐the‐shelf cell therapies additionally assures unwavering and predictable therapeutic efficacy.^[^ ^80^ ^]^ In this article, we will undertake a comprehensive investigation into off‐the‐shelf CAR products from three distinct procurement channels (**Table** **3**).

**Table 3:** Comparison of characteristics for ex vivo CAR products and three kinds of off‐the‐shelf CAR‐engineered immune cells.

Characteristics	Autologous CAR products	Allogeneic UCAR products	Allogeneic UCAR products	In vivo CAR products
Manufacturing process	Complex workflows; CAR‐ CAR‐engineered cells are induced in ex vivo settings; delay from leukapheresis to CAR‐engineered cells refusion; difficult to scale‐up production and preservation	More complex; apart from CAR transduction, additional genetic editing is required to prevent GVHD; Scaled‐up industrialized production and cryopreservation	Relative complex; Reprogram somatic cells into iPSCs, followed by inducing directed differentiation into immune cells and conducting CAR engineering; Scaled‐up industrialized production and cryopreservation	Simplified logistics; CAR‐ CAR‐engineered cells are induced in in vivo settings; engineering vectors are designed for scaled‐up industrialized production and preservation
Manufacturing conditions	GMP conditions	GMP conditions	GMP conditions	Non‐GMP conditions
Preparation period	Long (7–22 days)	Long but off‐the‐shelf	Long but off‐the‐shelf	Short (1–3 days), off‐the‐shelf
Persistence	Months to years; lengthy in vitro culture makes cells prone to exhaustion	Limited, weeks to months	Enhanced, months to years	Days to years; depending on the types of gene payloads
Redosing	Limited by autologous immune cells	Easy; anytime if required	Easy; anytime if required	Easy; only a single injection of engineering vectors
Significance for clinic and patient accessibility	Autologous; poor	Off‐the‐shelf; strong	Off‐the‐shelf; strong	Off‐the‐shelf; strong
Implementation cost	Currently very expensive	Expected to be acceptable	Expected to be acceptable	Moderate cost; relatively cheap
Main issues and risks	Highly relying on the quality and quantity of autologous immune cells; insertion mutation; CRS; ICANS; other CAR therapy‐related adverse reactions	Limited in vivo persistence and expansion; CRS; ICANS; GVHD; other CAR‐related adverse reactions	Risk of tumorigenicity, abnormal cell proliferation, and genetic instability; CRS; ICANS; low risk of GVHD; other CAR therapy‐related adverse reactions	Highly relying on the quality and quantity of autologous immune cells; immunogenicity and safety of engineering vectors; risk of off‐target transfection of other cells; CRS; ICANS; other CAR therapy

### 6.1 Allogeneic Universal off‐the‐shelf CAR‐Based Immunotherapy

**Scheme 5:**
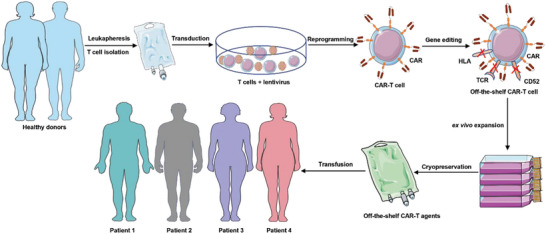
Schematic of the manufacturing logistics of off‐the‐shelf universal allogeneic CAR‐Ts. The workflow includes healthy donor leukapheresis; subsequent T‐cell isolation and activation; lentivirus transduction to induce expression of CARs; and further CRISPR/Cas9 gene editing to eliminate HLA, αβTCR, and CD52 molecules. The process ultimately yields universal CAR‐Ts and circumvents GVHD. The CAR‐T cells can then be amplified to make an off‐the‐shelf cell formulation, which is cryopreserved, shipped to hospitals, and ultimately transfused into cancer patients.

Given the limitations of ex vivo‐engineered autologous CAR drugs, the focus of recent efforts has shifted toward off‐the‐shelf allogeneic products generated based on universal allogeneic CAR‐immune cells from healthy donors,^[^ ^78^, ^81^ ^]^ which would allow immediate transfusion into cancer patients, preventing treatment delay for aggressive diseases. Allogeneic universal CAR‐immune cell technology is based on gene modification of immune cells isolated from healthy persons to express CARs, which overcomes the problem that some patients have insufficient endogenous immune cells. Distinct from autologous CAR‐Ts, allogeneic universal CAR (UCAR) products must undergo further gene editing to avoid GVHD caused by major MHC mismatch between the donor and the recipient^[^ ^81^ ^]^ (**Scheme** **5** and Table 3).

Allogeneic UCAR products offer the potential for utilizing off‐the‐shelf products in the treatment of r/r malignant neoplasms, and thereby the clinical trials involving the application of universal allogeneic CAR‐T cells are witnessing a surge of remarkable magnitude. Hu et. al developed CRISPR‐based universal off‐the‐shelf CD19/CD22 dual‐targeted CAR‐T cells as a novel therapeutic modality for r/r ALL. In this study, they knocked out TRAC region and CD52 gene to avoid GVHD. On day 28 after infusion, they achieved a CR rate of 83.3%, without dose‐limiting toxicity, ICANS, or genome editing‐associated adverse events.^[^ ^82^ ^]^ Other efforts against BCMA+ r/r multiple myeloma,^[^ ^83^ ^]^ CD19+ children with refractory B cell leukemia,^[^ ^84^ ^]^ and CD7+ ALL^[^ ^85^ ^]^ have been reported in recent years.

Evidently, there exist limitations to these off‐the‐shelf products. Firstly, excessive gene manipulation increases the risk of insertional mutations and further increases the technical demand. In addition, allogeneic UCAR‐T cells have been present constrained in vivo persistence and expansion in clinical settings. This challenge can be addressed by ameliorating host rejection responses or mitigating the immunogenicity of infused cells.^[^ ^81^ ^]^

### 6.2 Human‐Induced Pluripotent Stem Cells (iPSCs)‐derived off‐the‐shelf CAR Products

**Scheme 6:**
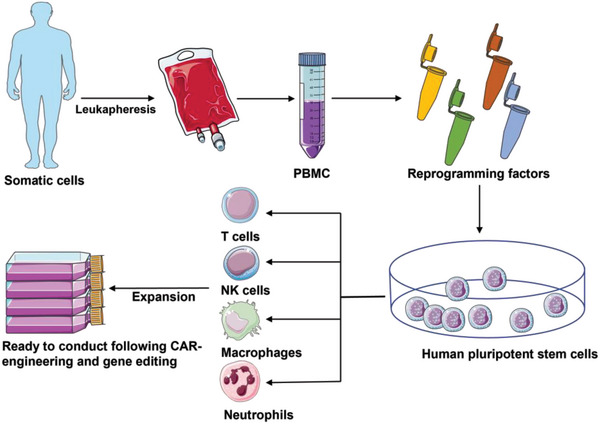
The establishment workflows of iPSCs‐derived immune cells. PBMC as the most common somatic cell that is isolated either from autologous or allogeneic donors via leukapheresis. Through the introduction of defined transcription factors, PBMC would be reprogrammed into human pluripotent stem cells. Subsequently, by virtue of cytokines and stromal cells, these iPSCs could be directed differentiation as either T cells (OP9‐DL1, OP9‐DLL4, and MS5‐DLL4), NK cells (IL‐15, IL‐7, IL‐3, and FIT3L), macrophages (IL‐3, M‐CSF, G‐CSF, and GM‐CSF), or neutrophils (FITSL, SCF, IL‐6, IL‐3, GM‐CSF, G‐CSF, and Am580). Then the obtained immune cells experience significant expansion and would be ready to conduct following CAR‐modification and gene editing to manufacture CAR products.

Other alternative off‐the‐shelf technologies, such as siPSCs‐derived CAR‐immune cells, have been gradually introduced; iPSCs theoretically could serve as an unlimited source of CAR‐immune cells because of the infinite proliferation potential (Table 3). The induction and reprogramming processes are exceptionally cumbersome, and the establishment processes of iPSCs‐derived immune cells are as follows (**Scheme** **6**). The most recent studies of this attractive technology have involved in generation of various types of CAR‐immune cells. A research group harnessed primary CD62L+ naive T cells and memory T cells (T_N/MEM_) enriched from the peripheral blood of healthy human donors, and subjected them to reprogramming via transduction of plasmids encoding KLF4, SOX2, OCT‐4, C‐MYC, and LIN28, along with P53 shRNA. Subsequently, these iPSCs were further transduced with a lentivirus carrying CD19 CAR and ultimately directed differentiation toward CD19 CAR‐T cells through a specialized 3D‐organoid culture system.^[^ ^86^ ^]^ Moreover, the iPSC‐derived CD19 CAR‐T cells exhibited robust in vivo antitumor efficacy, prolonging the survival of mice harboring CD19+ human tumor xenografts. Inspiredly, another group delineated the foundational concept and fabrication platform for generating CAR‐NK cells from iPSCs at the single‐cell clone level.^[^ ^87^ ^]^ They derived iPSC‐derived CD19 CAR‐NK cells from CD3−CD56+CD16+ NK cells isolated from healthy neonatal UCB. The introduction of three episomal plasmids encoding defined transcription factors, along with an additional plasmid encoding transient EBNA‐1, augmented the reprogramming efficiency. Given the prominent infiltrative ability of macrophages within solid tumors, there has been notable interest in iPSC‐derived CAR‐MΦ. Zhang et al. acquired iPSCs from healthy donor PBMCs, followed by CAR engineering and directed differentiation into CAR‐MΦ (CAR‐iMac).^[^ ^88^ ^]^ The CAR‐iMac demonstrated antigen‐dependent phagocytic activity and anticancer cell functionality both in vitro and in vivo. Furthermore, there was an antigen‐dependent augmentation observed in the expression of M1 pro‐inflammatory cytokines. Besides, scientists have encouragingly shifted this promising paradigm for CAR‐neutrophils (CAR‐N). Chang and colleagues achieved the stable generation of neutrophils from iPSCs through precise signal modulation. Employing CRISPR‐Cas9‐mediated homologous recombination, they ingeniously inserted three distinct glioblastoma (GBM)‐targeting CARs into iPSCs.^[^ ^89^ ^]^ Notably, their investigations highlighted the superior efficacy of the composite CLTX (a GBM‐targeting peptide)‐T‐CAR in enhancing the antitumor cytotoxicity of iPSC‐derived neutrophils. These cells exhibited classical neutrophil phenotypes and selectively killed tumor cells through membrane‐associated MMP2‐mediated interaction with GBM. Intriguingly, in an in vivo setting, CLTX‐T‐CAR neutrophils, upon injection, significantly curbed tumor growth and extended survival duration in an orthotopic GBM xenograft model, surpassing wild‐type neutrophils, NK cells, and CAR‐NK cells.

However, despite promising prospects, there are several potential issues that warrant cautious evaluation. First of all, the heterogeneity of iPSC remains to be overcome in different iPSC lines that lead to variation of differentiation deficiency.^[^ ^90^ ^]^ Secondly, due to their robust proliferative capacity and pluripotent differentiation potential, iPSCs may harbor genetic variations that could be magnified during cellular differentiation and expansion, potentially compromising the stability and safety of cellular therapies and even inciting oncogenicity. Thirdly, CAR‐immune cells derived from iPSCs may exhibit immunogenicity, rendering them susceptible to recognition and attack by the host immune system, potentially resulting in cellular clearance and declined therapeutic efficacy.

### 6.3 In Vivo Programming for CAR‐Based Gene Therapy Strategies

**Scheme 7:**
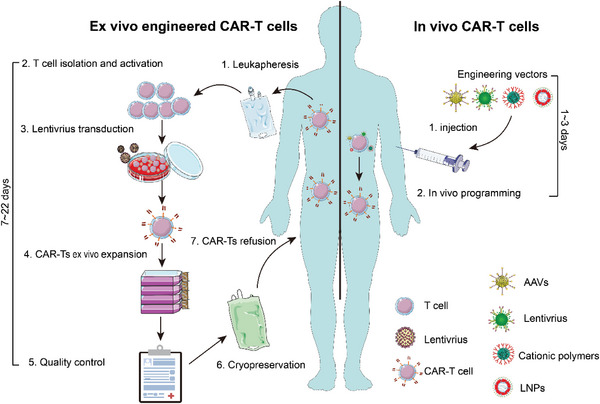
Schematic of the comparison of manufacturing of ex vivo and in vivo CAR‐T cells.

One of the latest strategies for off‐the‐shelf CAR technology for clinical treatment is in vivo generation of CAR‐modified immune cells, for which the CAR gene is precisely delivered to specific immunocytes in vivo by specifically designed off‐the‐shelf vectors.^[^ ^91^ ^]^ An ideal CAR gene delivery vector should be based on the following principles: (1) the target receptor is the main factor that needs to be considered, as it determines the ability to selectively bind to immunocytes for further in‐site reprogramming and prevents off‐target gene alteration in complex in vivo environments; (2) the gene payloads need to be internalized into immune cells and elicit durable or transient expression of the CAR protein to generate tumoricidal activity; (3) the preparation workflow needs to be simple and scalable to industrialized production, the formulation needs to be stable while in storage for long periods of time for the formulation to serve as an off‐the‐shelf therapeutic drug, and the cost needs to be low to be affordable for the majority of patients; and (4) the therapeutic vectors need to have a good safety profile. Several engineering vectors based on modified viral vectors and targeted nanoparticles (NPs) have been leveraged for induction of CAR‐T and CAR‐MΦ therapy in vivo. Below, we provide a brief overview of the current vectors used in delivery systems. (Table 3 and **Scheme** **7**)

### 6.4 Engineering Viral Vectors

Routine in vitro gene editing of CAR‐Ts relies largely on recombinant viral vectors, including γ‐retroviral vectors,^[^ ^9b^ ^]^ lentiviruses,^[^ ^92^ ^]^ adenoviruses, and AAV.^[^ ^93^ ^]^ Among these vectors, lentivirus vectors (LVs) can be used to efficiently transfect nondividing and dividing cells to transfer large exogenous gene fragments and maintain stable and durable transgene expression.^[^ ^94^ ^]^ Craig M. Rive et al. reported that simple intravenous infusion of replication‐incompetent glycoprotein G of vesicular stomatitis virus (VSV‐G)‐pseudotyped lentiviral vectors carrying an anti‐CD19 CAR transgene with either an FMC63 (human) or ID3 (murine) anti‐CD19 scFv into wild‐type mice effectively transfected murine T cells in vivo, resulting in CAR‐T proliferation and subsequent B‐cell ablation.^[^ ^95^ ^]^

**Scheme 8:**
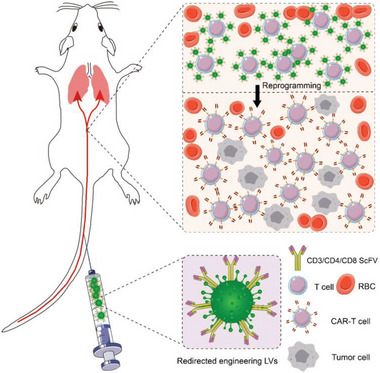
Schematic of in vivo reprogramming of CAR‐T cells with engineered LVs. LVs with altered tropism are engineered with target receptors (CD3/CD4/CD8 ScFv). Upon a single systemic administration, the LVs selectively recognize and bind to T cells, followed by subsequent transduction of the CAR gene and reprogramming of CAR‐Ts in vivo, generating T cells that specifically recognize and destroy tumor cells.

However, nontargeted VSV‐G‐pseudotyped lentivirus particles should also be considered. VSV‐G present on the envelope of pseudotyped LVs binds to low‐density lipoprotein receptor (LDLR) on the surface of most cells and mediates cell entry.^[^ ^96^ ^]^ Thus, VSV‐G determines the broad tropism of LVs and leads to their superior transduction potency in various mammalian cells, which is the theoretical basis of in vitro generation of CAR‐Ts by LVs. As expected, this natural tropism may, in turn, contribute to extensive off‐target transfection in in vivo reprogramming settings, in which the vectors are expected to deliver the CAR transgene to predetermined immune cells, not other cells, in a highly selective manner, avoiding adverse side effects for patients. Therefore, for in vivo generation of CAR‐engineered immunocytes, ideal engineering LVs should lack extensive tropism and possess the equipment of targeted receptors, ensuring specific recognition and binding of target cells (**Scheme** **8**). Lentiviruses pseudotyped with distinct envelope proteins such as paramyxoviruses, Sendai viruses, and alphaviruses, which contain separate envelope glycoproteins for binding and fusion and therefore possess decreased tropism,^[^ ^97^ ^]^ can target T cells with specific receptors. Several groups have pioneered the design of more advanced modified LVs for in vivo transduction and induction of CAR‐Ts.

Christian J. Buchholz and his groups have reported the generation of genetically modified CD8‐specific scFvs on Nipah virus (NiV)‐based LVs (CD8‐LVs), and upon a single systemic administration of CD8‐LVs, CD19‐reactive CD8+ CAR‐Ts were generated in humanized mice, accompanied by selective B‐cell depletion and CRS.^[^ ^98^ ^]^ In the subsequent study, they further developed CD3‐targeted LVs that activated T cells and induced their proliferation during the transduction process without the prior activation that is needed for traditional VSV‐G‐LVs, allowing gene transfer to be performed with unprocessed blood and further generating CAR‐Ts directly in vivo to eliminate endogenous B cells.^[^ ^99^ ^]^

Based on their encouraging results, this group designed additional engineered LVs specific to CD4+ and CD8+ T‐cell subsets for in situ gene editing, and in a CD19+ Raji lymphoma mouse model, they revealed that mice administered CD4‐LVs showed more powerful tumor suppressive effects than mice injected with CD8‐LVs alone or a mixture of CD8‐LVs and CD4‐LVs, suggesting that CD4+ CAR‐Ts may be superior to CD8+ CAR‐Ts.^[^ ^100^ ^]^ Similarly, in a CD19+ Nalm‐6 leukemia NOD‐scid‐IL2Rγ^null^ (NSG) mouse model humanized with both human peripheral blood mononuclear cells (huPBMCs) and human CD34+ hematopoietic stem cells, Christian J. Buchholz and his colleagues used a single injection of CD8‐LVs to completely eradicate tumor cells.^[^ ^101^ ^]^ Remarkably, these in vivo CAR‐engineered immunocytes contained not only T cells but also CD8+ NK cells (NK and NKT cells), suggesting that they likely contributed to additional tumor cell clearance. However, the human immune system in vivo is far more complex than the humanized mouse model described above. Compared with the common humanized NSG (huNSG) model, which is generated by transgenic expression of stem cell factor (SCF), GM‐CSF, and IL‐3, NSG‐SGM3 mice reconstituted with human stem cells (huSGM3 mice) develop elevated numbers of human myeloid cells^[^ ^102^ ^]^ and thus more closely mimic the human immune cell composition, and this mouse strain has been utilized to generate preclinical CAR‐T therapy‐associated CRS and neurotoxicity models.^[^ ^103^ ^]^ To investigate the in vivo generation of CAR‐Ts in the presence of human myeloid cells, this group also evaluated the huSGM3 mouse model for in vivo CAR‐T induction with CD4‐LVs and CD8‐LVs,^[^ ^104^ ^]^ and they showed that in vivo CAR‐T generation in mice with an intricate humanized immune system was less efficient than that in the huNSG model above reported owing to nonspecific phagocytosis of LVs by macrophages. As such, and given that phagocytosis could be prevented by producing LVs in cells lacking MHC‐I and overexpressing CD47,^[^ ^105^ ^]^ the team produced shielded CD4‐LV^sh^ and CD8‐LV^sh^ particles in an optimized packing cell line, β2M^−/−^ CD47^high^ HEK293T cells; these phagocytosis‐shielded targeted LVs achieved greatly improved in vivo transduction.

Redirecting lentiviral particles is vital for enabling cell type‐specific in vivo gene delivery. Unlike Christian J. Buchholz's genetic strategies for redirection, nongenetic retargeting strategies may be better and more adaptable. Another group redirected lentiviruses expressing a mutant Sindbis virus envelope protein (Sindbis pseudotyped lentiviral vector, SINV) with a bispecific antibody binder that can bind the mutant E2 glycoprotein on SINV‐LVs and CD3 on T cells.^[^ ^106^ ^]^ As hypothesized, this redirected lentiviral system had exceptional specificity and efficiency, with a single dose of the virus delivered to mice engrafted with huPBMCs generating CD19‐specific CAR‐Ts that markedly suppressed the growth of a preestablished B‐cell tumor xenograft.

**Scheme 9:**
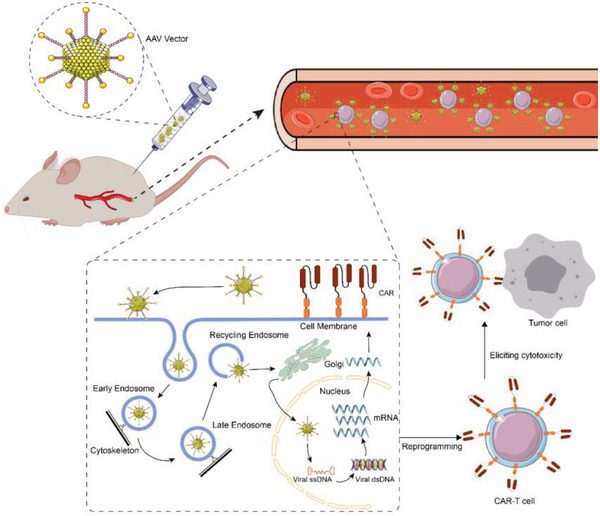
Schematic of in vivo generation of CAR‐Ts by AAV vectors carrying CAR genes. AAV vectors are systemically intraperitoneally injected into the body, and the AAV transfects T cells. The AAV binds to specific receptors on the cell surface and activates intracellular signaling pathways, which triggers AAV entry into the cell through receptor‐mediated endocytosis. With the assistance of endosomes, Golgi bodies, and other organelles, the AAV enters the nucleus, followed by viral disintegration. AAV single‐stranded DNA (ssDNA) is copied into double‐stranded DNA (dsDNA), and the CAR gene is delivered into the cell nucleus. Thus, T cells are edited into tumor‐specific CAR‐Ts in vivo, which are capable of eliciting specific cytotoxicity against tumor cells.

In addition to LVs, AAVs are emerging as a new generation of synthetic biology‐based engineering vehicles that can deliver the CAR transgene to specific immunocytes in vivo. In contrast to lentivirus‐mediated gene transfer, which results in integration into the host cell genome (which carries the risk of insertion mutations), AAVs mediate transient transfection with higher safety and lower immunogenicity.^[^ ^107^ ^]^ AAVs are not currently known to cause any human diseases. Increasing numbers of applications of AAV vectors in the clinic as in vivo gene therapy are showing their promise for both genetic disorders and complex diseases.^[^ ^108^ ^]^ Xilin Wu et al. were the first group to try using AAV‐DJ (type 2/type 8/type 9 chimeras) to edit CAR‐Ts in vivo^[^ ^109^ ^]^ (**Scheme** **9**). The researchers furnished the AAV vector with pDNA encoding a third‐generation CD4‐CAR, and upon single infusion into a humanized NOD.Cg‐Prkd^cscid^ Il2rg^em26^/Nju HuPBL (NCG‐HuPBL) mouse model of human CD4+ T‐cell leukemia, the AAV reprogrammed sufficient numbers of potent in vivo CD4‐reactive CAR‐Ts to result in tumor eradication. One major limitation of this work is the specificity of the AAV, and this group did not evaluate CAR expression in hematopoietic cells, tumor cells, or other immune and nonimmune cells. The strategy may result in the development of acquired resistance to the CAR‐T therapy and tumor immune escape if the CAR gene is unintentionally inserted into leukemia cells, as this would result in the antigen epitope being shielded from recognition by the CAR, as previously reported in a clinical trial.^[^ ^110^ ^]^ Despite this proof‐of‐concept of AAV‐generated CAR‐Ts in vivo, further efforts are still needed to develop novel AAV vectors carrying CAR genes that can exclusively target T cells.

For instance, designing AAV capsids with retargeting receptors via genetic manipulation and nongenetic modification or identifying AAV variants for efficient T‐cell transduction^[^ ^111^ ^]^ may accelerate preclinical research and clinical translation of AAV‐based strategies for the delivery of CAR genes to generate CAR‐T therapy in vivo. Preclinical studies of viral vectors carrying CAR genes for editing of CAR‐Ts in vivo are summarized below (**Table** **4**).

**Table 4:** Overview of viral vector‐based CAR‐T production in vivo in preclinical studies.

Vector	Glycoprotein pseudotype	Redirection receptor	Delivered gene	Dosing, administration route, and transfection efficiency	Target cell	Mouse/Tumor model	Reference
LVs	VSV‐G	None	FMC63/1D3‐CD19‐CAR‐GFP	2 × 10^7^ or 2 × 10^6^ IU; i.v.; 13.5% ± 0.58% in peripheral blood	Endogenous B cells	Wild‐type C57BL/6 mice	[95]
LVs	NiV	hCD8	hCD19‐28ζ‐CAR	2 × 10^6^ TU; i.p.; 30–50% in peritoneal fluid, 10–35% in spleen, and 5–30% in blood	Raji	NSG mice engrafted with CD34+ cells/B‐cell lymphoma	[98]
		hCD8	hCD19‐28ζ‐CAR	2 × 10^6^ TU; i.v.; CAR‐T percentage ranging between 1.5 and 14% of all CD8+ lymphocytes	Endogenous B cells	NSG mice engrafted with CD34+ cells	
LVs	NiV	hCD3	hCD19‐28ζ‐CAR	2 × 10^11^ particles; i.v.; maximum CAR‐T percentage in peripheral blood > 20%	Endogenous B cells	NSG mice engrafted with CD34+ cells	[99]
LVs	Measles virus	hCD4	hCD19‐28ζ‐CAR	4 × 10^10^ particles; i.p.; CAR‐T percentage 30%–65% in the peritoneum, spleen, and blood	Endogenous B cells	NSG mice engrafted with huPBMCs	[100]
		hCD4, hCD8	hCD19‐28ζ‐CAR	1× 10^11^ CD4‐LV particles, 2.5 × 10^11^ CD8‐LV particles, 5 × 10^10^ CD4‐LV particles and 1.25 × 10^11^ CD8‐LV particles (MIX); i.v.; CAR‐T levels in some particular animals reached >40% of CD4+ and CD8+ T cells	Nalm6‐Luc	NSG mice engrafted with huPBMCs/B‐cell leukemia	
		hCD4, hCD8	hCD19‐28ζ‐CAR	4 × 10^10^ particles; i.v.; some mice displayed 71% CAR+ cells within CD4+ lymphocytes in blood	Endogenous B cells	NSG mice engrafted with CD34+ cells	
LVs	NiV	hCD8	hCD19‐28ζ‐CAR	2.5 × 10^11^ particles; i.v.; 5%–12% of cells isolated from bone marrow, spleen, and blood on day 14 were CAR+CD3+CD8+ cells	Nalm6‐Luc	NSG mice engrafted with huPBMCs/B‐cell leukemia	[101]
LVs	NiV	hCD4, hCD8	hCD19‐28ζ‐CAR	2.4 × 10^11^ CD4‐LV particles, 1.2 or 2 × 10^11^ CD8‐LV particles or 1.6 × 10^11^ CD4‐LV particles and 7.6 × 10^10^ CD8‐LV particles (MIX); 8.7 × 10^10^ CD4‐LV or CD4‐LV^sh^ particles, 1.4 × 10^11^ CD8‐LV or CD8‐LV^sh^ particles; i.v.; the maximum CAR+ T cell percentage in peripheral blood 11%	Endogenous B cells	HuSGM3 mice engrafted with CD34+ cells	[104]
LVs	SINV	hCD3	hCD19‐28ζ‐CAR	5 × 10^10^ particles; i.v.; 0.2%–1.6% CAR+CD3+ T cells in peripheral blood	BV‐173‐Luc	NSG mice engrafted with huPBMCs/B‐cell leukemia	[106]
AAV‐DJ	None	None	hCD4‐28‐4‐1BBζ ‐CAR	1 × 10^11^ or 2 × 10^11^ vg; i.p.; 10%–35% CAR+CD8+ T cells and 10%–60% CAR+CD8+ T cells in peripheral blood	Endogenous CD4+ T cells and MT‐2‐Luc	NCG mice engrafted with huPBMCs/T‐cell leukemia	[109]

However, although the success of viral vector‐mediated CAR‐T production in vivo has provided new insights for novel generations of CAR‐T immunotherapy, successful clinical translation is still a long way off. First, the stability and durability of viral particles in vivo need to be further investigated. Viral particles, as foreign particles, have high immunogenicity and innate and adaptive immune responses to these particles reduce the efficacy and stability of in vivo gene transfer, constituting substantial obstacles to clinical translation and wider application in patients.^[^ ^112^ ^]^ For example, 50%−90% of humans have been exposed to AAVs, and ≈50% of AAVs infections result in the development of anti‐AAVs capsid‐neutralizing antibodies,^[^ ^113^ ^]^ commonly known as preexisting vector immunity. Additionally, vaccination with AAVs vectors can also elicit anti‐vector immunity.^[^ ^114^ ^]^ When it binds with injected engineering AAVs vectors, this preexisting antibody can significantly reduce the efficacy. Further investigation of other rare or nonhuman viral particles and novel engineered viral vectors that can circumvent, suppress, or manipulate the immune response should increase the vector choices, ideally resulting in sustained expression of CAR cargo in vivo and immune tolerance of the vectors. Second, the safety of viral vectors and in vivo CAR‐Ts needs to be further evaluated. Lentivirus‐mediated gene transduction carries the risk of insertion mutations,^[^ ^10^ ^]^ and the process will be more complex and unpredictable in in vivo settings. Furthermore, CD8‐LVs are used to generate in vivo CAR‐T‐induced CRS,^[^ ^98^ ^]^ similar to what occurs in patients treated with CAR‐T immunotherapy in the clinic.^[^ ^115^ ^]^ Accordingly, more deeply studying the mechanisms of insertional mutagenesis specific to intrinsic target sites^[^ ^116^ ^]^ and performing clinical trials on in vivo CAR‐T therapy might improve the prediction and management of adverse events. Finally, in vivo viral vectors necessitate taking count of equity simultaneously between stable delivery of gene cargos and meticulous modification of targeting moiety, thereby demanding increasingly intricate and sophisticated designs. This represents a technical challenge for the future transition of in vivo off‐the‐shelf CAR products from laboratory settings to clinical applications.

### 6.5 Engineering Biomaterials

In recent years, preclinical research on strategies leveraging therapeutic biomaterials has increased worldwide, with settings ranging from regenerative medicine and tissue engineering to oncotherapy and medical imageology.^[^ ^117^ ^]^ Previous studies have utilized biomaterials as vectors to deliver CAR gene cargo to T cells and macrophages and produce CAR‐Ts and CAR‐MΦs ex vivo.^[^ ^118^ ^]^ Unsurprisingly, increasing interest has led to the development of engineering biomaterials amenable to in vivo production of CAR‐engineered immunocytes. Biomaterials can be designed as targeted nonviral vehicles for CAR gene delivery in vivo and the systemic generation of CAR‐bearing immune cells, as well as to have high biocompatibility to accommodate recombinant gene delivery vectors mediating in situ transduction locally.

Non‐viral vectors as a means for gene delivery, such as cationic polymers, lipids, inorganic nanoparticles, and combinations of different agents, have attracted much attention, demonstrating great application potential due to their lower cytotoxicity, immunogenicity, and mutagenesis rate in comparison to viral vectors.^[^ ^119^ ^]^ Likewise, biomaterials for T‐cell engineering generated through novel methods are being increasingly recognized as reliable alternatives for next‐generation CAR‐T‐cell manufacturing in vitro and in vivo^[^ ^13a^ ^]^ (**Table** **5**).

**Table 5:** Overview of engineered biomaterials used for vector‐mediated CAR‐T and CAR‐MΦ production in vivo in preclinical studies.

Biomaterial	Targeting receptor	Payload	Delivered gene	Product	Dosing, administration route and transfection efficiency	Target cell	Mouse/Model	Reference
Cationic polymers								
PBAE NPs	mCD3	pDNA	iPB7‐mCD19‐4‐1BBζ‐CAR	CAR‐T	3 × 10^11^ particles/day, repeat for five days; i.v.; 7.1% ± 1.7% CAR+ cells among CD3+ T cells at day 24	Eµ‐ALL01	^a)^Albino B6/Leukemia	[122]
PBAE NPs	mCD3	mRNA	mCD19‐28ζ‐CAR	CAR‐T	6 weekly doses, 50 µg mRNA/dose i.v.; 10% ± 4.3% CAR+ cells among CD8+ T cells on day 2 after the first dose	Eµ‐ALL01	Albino B6/Leukemia	[124]
hCD8	mRNA	hCD19‐28ζ‐CAR	CAR‐T	6 weekly doses, 50 µg mRNA/dose; i.v.; 8.1 ± 1.9%	Raji‐Luc	huNSG/Lymphoma
mCD8	mRNA	h^b)^ROR1‐4‐1BBζ‐CAR	CAR‐T	4 weekly, 3 daily doses; 15 mg mRNA/dose; i.v.; 6.34% CAR+CD45+ T cells at day 11 after treatment	LNCaP C42‐Luc	huNSG/Prostate carcinoma
MPEI NPs	mCD206	pDNA	piggyBac‐mALK‐ IFN‐γ‐CAR	CAR‐MΦ	25 µg pDNA; i.v.; 82.0 ± 27.0% CAR+ macrophages in tumor tissues	Neuro‐2a	A/J/Neuroblastoma	[127]
	LNPs							
CD5‐LNPs	mCD5	mRNA	mFAP‐28ζ‐CAR	CAR‐T	10 µg LNPs; i.v.; 17.5–24.7% CAR+ T cells on day 2 after LNP injection	Activated endogenous fibroblasts	C57BL/6J/Cardiac injury	[130]
AntiCD3‐LNP/CAR19 + shIL6	hCD3	pDNA	iPB7‐IL6shRNA‐ hCD19‐28/4‐1BBζ‐CAR	CAR‐T	5 µg pDNA/g of mouse weight; i.v.; mean 74.6% CAR+ cells among CD3+ T cells at day 21	CD19‐K562, Raji‐Luc	huNSG/Lymphoma	[132]
Macroencapsulation devices								
MASTER	None	γ‐retrovirus	hCD19‐28ζ‐CAR	CAR‐T	1 × 10^6^ PBMCs and concentrated retrovirus at an MOI of 2 were seeded in each scaffold; s.c. implant; 22% ± 1% CAR+ cells among T cells at day 3	Daudi‐FFLuc	NSG/Lymphoma	[134]
Nanoporter hydrogel superstructure	mCD206	pDNA	iPB7‐mCD68p‐mCD133 ‐CD3ζ‐CAR	CAR‐MΦ	2 mg NP/kg of mouse weight; ^c)^i.t.; the frequency of CAR‐MΦ in macrophages was 5.02 ± 1.2% on day 6 and gradually increased to 12.13 ± 1.39% on day 12	GL261/Patient‐derived primary CD133+ cells	C57BL/6J/Glioma, ^d)^huHSC‐NOG‐EXL/PDX of glioma	[135]

^a)^ Albino B6, B6N‐Tyrc/BrdCrCrl;

^b)^ ROR1, receptor tyrosine kinase‐like orphan receptor 1;

^c)^ i.t., intracavity injection;

^d)^ huHSC‐NOG‐EXL, human hematopoietic stem cells NOD.Cg‐Prkdcscid Il2rgtm1SugTg (SV40/HTLV‐IL3, CSF2)10‐7Jic/Ji.

**Scheme 10:**
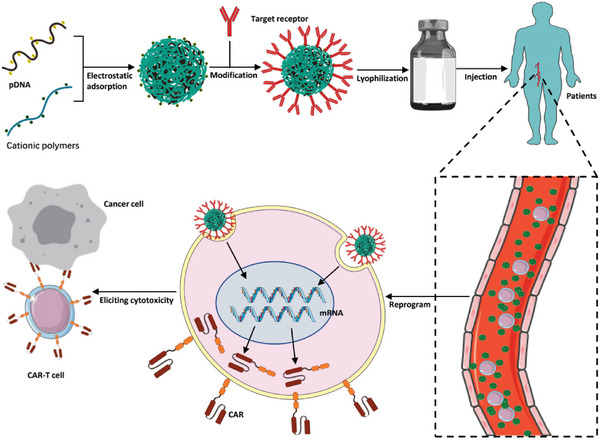
Schematic diagram of in vivo generation of CAR‐Ts by cationic polymers. pDNA encoding the CAR gene is condensed into cationic polymers in a nanocomplex via electrostatic interactions. Subsequently, the nanocomplex is further modified with the target receptor. After lyophilization and storage, these off‐the‐self agents can be redissolved and injected into cancer patients. In peripheral blood, the nonviral vectors bind to circulating T cells and convert them into CAR‐Ts.

Cationic polymers have been the most widely used nonviral gene delivery vectors owing to their excellent capacity to form polyplexes with DNA or RNA through electrostatic interactions.^[^ ^120^ ^]^ Modified cationic polymers engineered to carry targeting receptors may be a viable alternative in vivo delivery vehicles. Poly(β‐amino ester) (PBAE) is a cationic polymer synthesized from an acrylate and an amine by Michael addition.^[^ ^121^ ^]^ Matthias T. Stephan and collaborators were the first to describe a method to quickly edit circulating T cells in vivo with PBAE‐based DNA‐carrying NPs^[^ ^122^ ^]^ (**Scheme** **10**). The researchers first coupled anti‐CD3e f(ab′)2 fragments to the surfaces of NPs, which enabled selective T‐cell targeting. Furthermore, they functionalized the polymer with peptides containing microtubule‐associated sequences (MTASs) and nuclear localization signals (NLSs) to facilitate intracellular transport and nuclear import. To achieve persistent CAR expression, a PB transposon/transposase plasmid system encoding a murine CD19 CAR gene was coencapsulated into the carriers. In subsequent experiments, these nanocarriers efficiently bound circulating T cells and introduced the CAR gene into the nucleus, thereby bringing about long‐term tumor remission. Unlike viral‐ and DNA‐based strategies for protein expression, messenger RNA (mRNA) cannot be integrated into the host genome, and the induced expression is transient. Since the dawn of the era of mRNA vaccines for SARS‐CoV2 infection, mRNA technology has been a focus of research attention.^[^ ^123^ ^]^ In subsequent research, the group modified the nanocarriers to introduce in vitro‐transcribed CAR‐specific mRNA cargo into circulating lymphocytes.^[^ ^124^ ^]^ In mouse models of human leukemia and prostate cancer, repeated infusions of these nanocarriers induced the production of sufficient endogenous T cells expressing tumor‐specific CARs to cause tumor suppressive effects at levels similar to those generated by adoptive T‐cell therapy. Moreover, poly(ethylenimine) (PEI) and its derivatives are other cationic polymers that are viable alternatives for in vivo nucleic acid delivery.^[^ ^125^ ^]^ Mannose receptors (CD206) are reported to be overexpressed in M2 phenotype tumor‐associated macrophages (TAMs).^[^ ^126^ ^]^ Mikyung Kang and his coworkers used mannose‐conjugated poly(ethylenimine) (MPEI) as a gene delivery carrier to target macrophages.^[^ ^127^ ^]^ To induce persistent expression of CARs and a shift from immunosuppressive M2 macrophages to immunostimulatory M1 macrophages, the researchers utilized the piggyBac transposon system and fused an interferon‐γ (IFN‐γ) gene construct. Condensed pDNA encoding an anaplastic lymphoma kinase (ALK)‐specific CAR and IFN‐γ was condensed into MPEI in a nanocomplex (MPEI/pCAR‐IFN‐γ) via electrostatic absorption. Intravenous injection of MPEI/pCAR‐IFN‐γ in vivo successfully induced CAR‐M1 macrophages that were capable of CAR‐mediated cancer cell phagocytosis and antitumor immunomodulation and suppressed solid tumor growth. Collectively, this study established a proof‐of‐concept for the in vivo reprogramming of M2 phenotype TAMs in the TME into CAR‐engineered M1 macrophages via nonviral vector‐mediated gene delivery for the treatment of solid tumors.

**Scheme 11:**
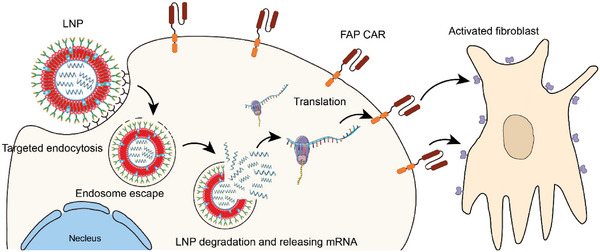
Schematic of the in vivo creation of transient FAP‐CAR‐Ts using CD5‐targeted mRNA‐carrying LNPs. mRNA‐carrying LNPs are targeted for endocytosis by circulating T cells. In the cytoplasm, the LNPs escape from the endosome and are degraded, releasing the mRNAs encoding the FAP‐CAR proteins. Subsequently, the T cells are transformed into CAR‐Ts targeting the marker FAP, which is overexpressed on activated fibroblasts.

Lipid nanoparticles (LNPs) are lipid vesicles with a homogeneous lipid core favoring drug encapsulation and have been extensively investigated for the delivery of nucleic acid drugs.^[^ ^128^ ^]^ To date, two mRNA‐LNP‐based vaccines have been marketed for combatting COVID‐19 with tremendous success,^[^ ^129^ ^]^ and thus, LNPs have recently gained unprecedented attention. Joel G. Rurik and others developed a modified mRNA encoding a CAR against fibroblast activation protein (FAP) (a marker of activated fibroblasts) in CD5‐targeted LNPs^[^ ^130^ ^]^ (**Scheme** **11**). This delivery system efficiently converted circulating T cells into FAP‐CAR‐Ts in a mouse model of heart failure, and FAP‐CAR‐Ts eliminated activated fibroblasts in a dose‐dependent manner similar to their virally engineered counterparts, alleviating fibrosis and restoring cardiac function after injury. It is worth noting that in this work, these in vivo‐reprogrammed CAR‐T cells were only transiently present, as the mRNA was not integrated into the genome. The strategy is likely to be accompanied by limited toxicities because fibroblast activation is ubiquitous in normal wound‐healing processes in many tissues as well as in inflammatory diseases^[^ ^131^ ^]^; furthermore, long‐lasting antifibrotic CAR‐Ts may increase the risk of “off‐target toxicity” in future trauma or inflammation. Other nucleic acid cargos have also been packaged in LNPs for in vivo CAR‐T production. Zhou et al. recently constructed an LNP encapsulating pDNA encoding interleukin 6 short hairpin RNA (IL‐6 shRNA) and a CD19‐CAR; the surface of this platform was also modified with an anti‐CD3 antibody.^[^ ^132^ ^]^ For durable expression of the CAR, MTAS‐NLS peptide and iPB7 transposons were also loaded in the LNP. This delivery system induced stable and specific transduction of endogenous T cells and generated IL‐6‐knockdown CAR‐Ts within 90 days to eradicate the tumor while also mitigating CRS in huNSG mice, exhibiting an antitumor effect equivalent to that of traditional CAR‐Ts manufactured ex vivo but with improved safety.

**Scheme 12:**
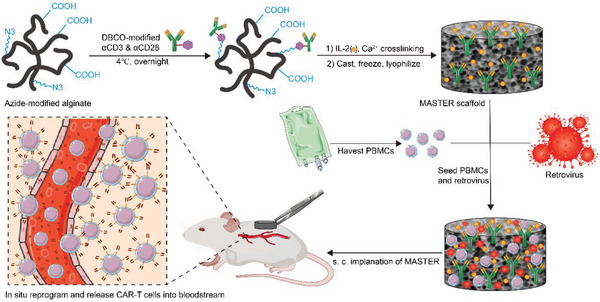
Schematic diagram of in vivo reprogramming of CAR‐Ts and their release into the bloodstream by the MASTER system. To prepare MASTER, azide alginate is incubated with DBCO‐modified anti‐CD3 and anti‐CD28 antibodies at 4 °C overnight. Recombinant human IL‐2 is then added, and the solution is stirred. Then, the resulting solution is vigorously stirred with an equal volume of 0.4% calcium gluconate, distributed into 24‐well plates for in vitro studies or 48‐well plates for in vivo studies, frozen at −20 °C overnight, and lyophilized. Finally, human PBMCs and γ‐retrovirus are seeded into the MASTER scaffold. When subcutaneously implanted in mice, MASTER provided the appropriate interface for viral vector‐mediated transduction and mediated the release of functional CAR‐Ts into the bloodstream.

Macroencapsulation devices such as membrane‐controlled release systems, 3D stents, microneedle array patches, and hydrogels have been utilized to enhance cellular therapies, and thus far, such systems have improved preclinical outcomes by supporting the mechanical and physiochemical milieu to maintain cell survival and proliferation and improve therapeutic functions.^[^ ^133^ ^]^ Biomaterials such as 3D scaffolds and hydrogels have porous properties that enable recombinant gene delivery vector encapsulation and cell accommodation. Two attempts with 3D scaffolds and hydrogels have recently been made to locally deliver CAR genes and reprogram CAR‐engineered immunocytes in situ. Pritha Agarwalla and his colleagues fabricated an implantable, multifunctional 3D alginate scaffold accommodating T cells and γ‐retroviral particles for T‐cell engineering and release (MASTER) that achieved in vivo CAR‐T‐cell manufacture and shortened processing time to a single day^[^ ^134^ ^]^ (**Scheme** **12**). Macroporosity was achieved in this scaffold through mild cryogelation to offer an interface for sufficient interaction between T cells and the retrovirus. Moreover, to promote T‐cell activation and transfection within the scaffold, anti‐CD3 and anti‐CD28 antibodies were covalently attached to alginate. After seeding of huPBMCs and γ‐retrovirus, upon subcutaneous implantation, MASTER served as a bioinstructive factory permitting CD19‐CAR‐T generation and expansion, resulting in the release of CAR‐Ts into the bloodstream, further suppressing distal tumor growth in a mouse xenograft model of lymphoma.

**Scheme 13:**
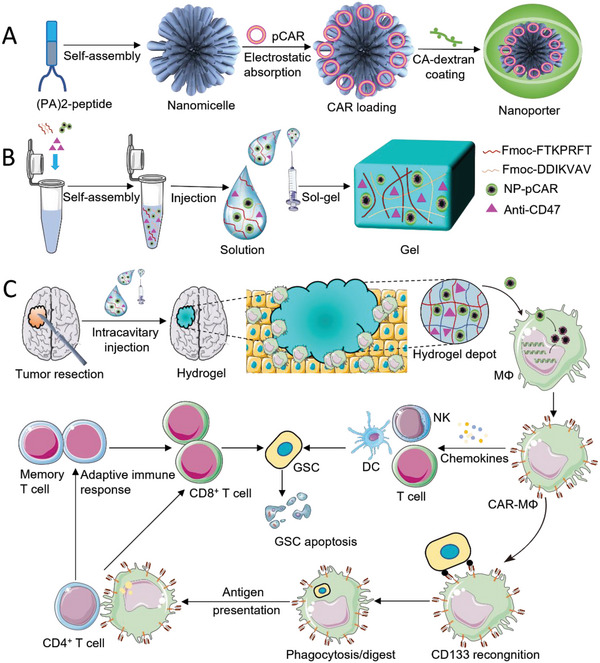
Schematic diagram of intracavitary generation of GSC‐specific CAR‐MΦs for clearing remaining tumor cells and preventing postoperative glioblastoma relapse. A) Schematic illustration of the preparation of pCAR‐laden nanoporters (NP‐pCAR). B) Schematic illustration of the self‐assembled peptide hydrogel for the encapsulation of NP‐pCAR and anti‐CD47 antibody. C) Schematic illustration of the locoregional generation of CD133‐specific CAR‐MΦs surrounding the tumor cavity by intracavity‐injected nanoporter‐hydrogel superstructure for clearing residual cancer cells and preventing postoperative GBM relapse by activating the adaptive immune response.

Another research team reported an intracavity‐injectable nanoporter‐hydrogel microencapsulation strategy that could generate CAR‐MΦs locally surrounding the cavity.^[^ ^135^ ^]^ This system was prepared by mixing brain extracellular matrix‐mimetic resultant hydrogel with a nanoporter synthesized by a nanomicelle self‐assembled by NLS peptide as the hydrophilic moiety and palmitic acid (PA) as the hydrophobic domain and was further coated with citraconic anhydride‐modified dextran (CA‐dextran) to achieve CD206 receptor targeting; pDNA encoding the MΦ‐specific CD68 promoter and a CD133‐CAR were also electrostatically adsorbed on the surface (**Scheme** **13**A,B). Subsequent in vivo experiments demonstrated that after intracavity delivery, this platform could introduce CD133‐CAR genes into the nuclei of postoperative macrophages and resident microglia to locoregionally generate CAR‐MΦs in situ in mouse models of GBM. Notably, these intracavitary CAR‐MΦs were able to hunt for and phagocytose glioblastoma stem cells (GSCs), eliminate residual GSCs, and prevent recurrence by priming endogenous adaptive antitumor immunity in the TME (Scheme 13C). Encouragingly, when an anti‐CD47 antibody was added, the effects of the CAR‐MΦ‐mediated in vivo tumor phagocytosis and induced adaptive immunity were further boosted, leading to robust tumor control and prolonging the survival time of all animals to >120 days.

In summary, we have described three types of representative biomaterial‐based vectors that can be used to create CAR‐Ts and CAR‐MΦs in vivo (Table 3). These biomaterial‐based platforms have high multifunctionality, diverse modifiability, and high drug‐loading properties and thus can be used to develop more versatile delivery systems to broaden the applicability of in vivo CAR‐engineered immunocyte therapy and further enhance the efficacy of CAR‐T immunotherapy. For example, altering the formulation and/or targeting moiety of cationic polymer‐based NPs and LNPs may enable selective binding to circulating NK cells, NKT cells, and γδT cells, which can then be transformed into CAR‐NKs, CAR‐NKTs, and CAR‐γδTs in vivo, respectively. Also, T‐cells with natural killer phenotype termed CIKs (mainly CD3+CD56+), refer to a heterogeneous population of mononuclear cells derived from peripheral blood, bone marrow, and umbilical cord blood. These individual nucleated cells are obtained through ex vivo cultivation (11–15 days), where they undergo stimulation with various cytokines, such as anti‐CD3 monoclonal antibodies, IL‐2, and IFN‐γ, to induce their cytotoxic activity.^[^ ^136^ ^]^ This promising immune effector cell population combines both, innate and adoptive immune responses, possessing potent antitumor activity similar to T lymphocytes and the MHC‐unrestricted tumor‐killing advantages of NK cells.^[^ ^137^ ^]^ The immunotherapy based on CAR‐CIK has been validated in several studies.^[^ ^3^, ^138^ ^]^ Through carefully designed formulations of biomaterial‐based carriers and delivering gene cargoes encoding CAR and stimulant cytokines, in situ generation and massive expansion of antigen‐specific CAR‐CIKs in vivo may be a novel source of off‐the‐shelf cellular agents.

Recently, these platforms have been combined with immune checkpoint inhibitors^[^ ^139^ ^]^ and stimulatory cytokines,^[^ ^140^ ^]^ as well as strategies to overexpress or knockout transcription factors that prevent or induce exhaustion^[^ ^141^ ^]^ to achieve robust tumoricidal immunity, longstanding persistence, and a favorable memory phenotype. Furthermore, factors reshaping the metabolic state of CAR‐Ts are another option in the context of a hostile TME.^[^ ^142^ ^]^ However, CRS and ICANS still limit the clinical application of CAR‐Ts.^[^ ^7a^ ^]^ As we discussed earlier, the application of CD8+ CD19‐CAR‐Ts generated from engineered LVs can result in signs of CRS,^[^ ^98^ ^]^ demonstrating that CAR‐T‐associated toxicity will still be a challenge in in vivo contexts. The LNPs created by Zhou et al discussed in this review silenced IL‐6 by coexpression of IL‐6 shRNA and CAR genes to overcome this.^[^ ^132^ ^]^ Thus, knocking out key factors or including an on‐off switch may improve the safety of this cell therapy. Furthermore, codelivery with corresponding pDNAs, mRNAs, and shRNAs or siRNAs could prevent immune hypofunction of CAR‐Ts in vivo and provide possibilities for alleviating toxicities.

Efforts are being made to commercialize mRNA‐LNPs for gene transfer technologies for in vivo CAR‐T induction, and Simnova and Orna Therapeutics have collaborated to develop engineered circular RNAs (oRNAs) expressing CARs and custom‐built LNPs designed to deliver oRNAs to cells of the immune system. Furthermore, Carisma's engineered macrophage technology has been combined with Moderna's mRNA and LNP technologies to develop in vivo CAR‐MΦ therapeutics for malignant tumor therapy. Hopefully, well‐established delivery strategies employing LNP‐based in vivo CAR‐engineered immunocytes will achieve clinical translation. Notably, based on clinical data from mRNA‐LNP vaccines, reported adverse events include localized and systemic anaphylaxis, and severe adverse reactions involving acute myocardial infarction, myocarditis, pericarditis, thrombosis, neurological complications, and autoimmune disorders.^[^ ^143^ ^]^ Therefore, thoroughly investigating and exploring the immunogenicity, potential toxicity, as well as short‐ and long‐term adverse events associated with LNP‐based in vivo CAR delivery vectors, could contribute to enhancing future clinical safety assessments and the formulation of responsive measures. Furthermore, other biomaterials including cationic polymers and macroencapsulation devices need further evaluation and deeper study before commercialization. For instance, their immunogenicity, biocompatibility, and pharmacokinetics need to be considered. As a final point, the intricate composition and formulation of CAR gene delivery vectors based on engineered biomaterials pose enormous technical challenges, underscoring the significance of interdisciplinary collaboration.

## 7 Conclusion and Future Prospects

Given the successful applications of CAR‐T therapy in oncology, the development of additional strategies with new technologies and improved ease of operation to reduce costs and increase accessibility is warranted. Continuous advances in broadening cell sources and engineering approaches have revealed new ways to improve the supply of CAR‐immune cells and simplify the manufacture of CAR products. The diverse sourcing strategies, encompassing autologous, donor‐derived, third‐party, and off‐the‐shelf cellular products, have unveiled a spectrum of cellular reservoirs with distinct attributes and potentials. These strategies have not only expanded the repertoire of therapeutic candidates but have also addressed limitations associated with cell availability and functionality. Moreover, ingenious engineering approaches have propelled the optimization of CAR‐based immunotherapies. Techniques such as genome editing, synthetic biology, and multi‐gene integration have enabled the tailoring of immune cells with enhanced persistence, specificity, and safety profiles. Concurrently, advancements in modular CAR designs, incorporation of costimulatory domains, and switchable CAR systems may have fine‐tuned the therapeutic response and mitigated adverse events, underscoring the remarkable progress achieved in refining CAR‐engineered immune cells.

At present, malignant neoplasms stand as the primary indications for CAR immunotherapies. However, CAR‐T techniques have been extended to treat diseases beyond cancer. For example, LNP‐based in vivo CAR‐Ts have been used as therapy for fibrotic disease.^[^ ^130^ ^]^ Strategies for other diseases are being developed, including senolytic CAR‐Ts (which can reverse senescence),^[^ ^144^ ^]^ engineered CAR‐Ts to target human immunodeficiency virus (HIV),^[^ ^145^ ^]^ and pulmonary aspergillosis,^[^ ^146^ ^]^ CD19‐targeted CAR‐Ts for the treatment of refractory systemic lupus erythematosus (SLE)^[^ ^147^ ^]^ and antisynthetase syndrome,^[^ ^148^ ^]^ chimeric autoantibody receptor T cells (CAAR‐T) for the treatment of pemphigus vulgaris^[^ ^149^ ^]^ and muscle‐specific tyrosine kinase myasthenia gravis (MuSK MG),^[^ ^150^ ^]^ and chimeric antigen receptor regulatory T cells (CAR‐Tregs) for the treatment of colitis^[^ ^151^ ^]^ and multiple sclerosis (MS).^[^ ^152^ ^]^ Therefore, in the future, expanding the applications of CAR immune cells from diverse sources and engineering approaches may result in enhanced research and development.

Looking ahead, further investigations are warranted to comprehensively elucidate the long‐term safety and efficacy of these advanced therapies. The development of standardized protocols for sourcing, engineering, and characterization of CAR‐modified immune cells will be instrumental in ensuring reproducibility and facilitating regulatory approval. Enhanced and collaborative efforts between medical centers, the biotechnology industry, pharmaceutical companies, and institutes of nanotechnology are poised to expedite the translation of these cutting‐edge approaches into transformative clinical interventions, potentially reshaping the landscape of cancer and other immune‐related diseases. As we navigate these frontiers, an exciting era of precision immunotherapy emerges, holding the promise of personalized, potent, and durable treatments for patients in need.

## Conflict of Interest

The authors declare no conflict of interest.
